# Clinical protection against caprine herpesvirus 1 genital infection by intranasal administration of a live attenuated glycoprotein E negative bovine herpesvirus 1 vaccine

**DOI:** 10.1186/1746-6148-3-33

**Published:** 2007-12-05

**Authors:** Julien Thiry, Maria Tempesta, Michele Camero, Elvira Tarsitano, Benoît Muylkens, François Meurens, Etienne Thiry, Canio Buonavoglia

**Affiliations:** 1 Department of Animal Health and Well-being, Faculty of Veterinary Medicine, University of Bari, 70010 Valenzano, Italy; 2 Department of Infectious and Parasitic Diseases, Virology and Viral Diseases, Faculty of Veterinary Medicine, University of Liège, 4000 Liège, Belgium; 3 Lymphocyte et Immunité des Muqueuses, IASP 311, UR 1282, National Institute of Agronomy Research (INRA), 37380 Nouzilly, France

## Abstract

**Background:**

Caprine herpesvirus 1 (CpHV-1) is responsible of systemic diseases in kids and genital diseases leading to abortions in goats. CpHV-1 is widespread and especially in Mediterranean countries as Greece, Italy and Spain. CpHV-1 is antigenically and genetically closely related to bovine herpesvirus 1 (BoHV-1). Taking into account the biological properties shared by these two viruses, we decided in the current study to assess the protection of a live attenuated glycoprotein E (gE) negative BoHV-1 vaccine against a genital CpHV-1 infection in goats.

**Results:**

The vaccine was inoculated intranasally twice three weeks apart followed by a subsequent CpHV-1 intravaginal challenge which is the natural route of infection in three goats. To analyse the safety and the efficacy of this marker vaccine, two groups of three goats served as controls: one immunised with a virulent CpHV-1 and one uninoculated until the challenge. Goats were clinically monitored and all sampling procedures were carried out in a blind manner. The vaccine did not induce any undesirable local or systemic reaction and goats did not excrete gE-negative BoHV-1. After challenge, a significant reduction in disease severity was observed in immunised goats. Moreover, goats immunised with either gE-negative BoHV-1 or CpHV-1 exhibited a significant reduction in the length and the peak of viral excretion. Antibodies neutralising both BoHV-1 and CpHV-1 were raised in immunised goats.

**Conclusion:**

Intranasal application of a live attenuated gE-negative BoHV-1 vaccine is able to afford a clinical protection and a reduction of virus excretion in goats challenged by a CpHV-1 genital infection.

## Background

The subfamily *Alphaherpesvirinae *includes a cluster of closely related ruminant viruses with bovine herpesvirus 1 (BoHV-1) as prototype [1]. BoHV-1, a major cattle pathogen, is typically responsible of infectious bovine rhinotracheitis (IBR) causing severe economic losses in livestock [2]. Since its isolation, several conventional vaccines have been developed. These vaccines usually prevented clinical signs and reduced the amount of excreted viruses. However, there was still a need for improvements in order to use them in control and/or eradication programmes [3]. Therefore, BoHV-1 marker vaccines comprising attenuated or killed mutants with a deletion in one of the non-essential genes (gE) were developed and eradication campaigns were initiated in many European countries. They have proven their safety and efficacy in the target bovine species since they are efficacious at reducing disease severity, virus shedding, and circulation in a population [4,5].

Caprine herpesvirus 1 (CpHV-1) is associated with two different syndromes in goats, a lethal systemic disease in kids [6,7] and a genital disease leading to balanoposthitis [8], vulvovaginitis [9] and abortion [10] in adults. These clinical signs and the virus presence in nasal, ocular, rectal and vaginal samples suggest both the venereal transmission as the principal virus entry route and infection persistence within herds [11,12]. The genital tropism of CpHV-1 was confirmed by the detection of viral DNA in sacral ganglia of latently infected goats [13]. According to serological investigations, the infection occurs worldwide with highest prevalences observed in Mediterranean countries [14-20]. However, the economical losses due to CpHV-1 infection are probably underestimated. To date, a classical inactivated vaccine has been developed [21,22], however, it can not be licensed since the market of veterinary medicinal products for minor species, like goats, is not economically profitable. Consequently, the control of this infection still relies on hygienic prophylactic measures [1].

BoHV-1 and CpHV-1 are antigenically and genetically closely related [1]. This relationship was originally demonstrated by serological assays [15,23-25] and lately by phylogenetic analysis [26-28]. These viruses are able to some extent to cross the species barrier and establish infection in heterologous animal species [29,30]. Experimental reactivation of latent infection of BoHV-1 in goats was successfully performed [31]. Moreover, a recent experiment showed that intranasal administration of a live attenuated gE-negative BoHV-1 vaccine in goats reduced the peak viral titre after a nasal CpHV-1 challenge and therefore afforded a partial cross-protection [32].

In the following study, it is hypothesised that an intranasal administration (of a bovine vaccine) could afford a protection against the clinical genital infection. Indeed, for many years, the upper respiratory mucosa has been proven to be suitable for vaccine delivery. The recent advances in the study of the mucosal immune system strengthen this mode of administration as being a very effective route for vaccination for both peripheral and mucosal immunity [33]. In human, nasal mucosa can serve as an efficient site for the induction of specific IgA and IgG responses in vaginal secretions [34,35]. The goat genital tract might employ similar homing mechanisms as those of the upper respiratory tract and therefore could receive primed immune cells from the nasopharynx-associated lymphoid tissue (NALT) [36]. Therefore, it was decided to investigate gE-negative BoHV-1 intranasal route of vaccination in goats with the aim to protect this species against CpHV-1 genital infection.

## Results

### Clinical and viral responses after intranasal immunisation

Goats immunised by intranasal inoculation with virulent CpHV-1 or gE-negative BoHV-1 vaccine remained in good general state of health. No signs of severe disease as anorexia, depression, oedema or lesions were observed. The gE-negative BoHV-1 immunisation did not induce any undesirable local or systemic reaction and goats did not show any clinical sign of disease. On the opposite, goats inoculated with CpHV-1 expressed mild clinical signs as hyperemia and nasal discharge. Based on the mean rectal temperature, the statistical analysis revealed significant differences between groups (p < 0.005). From day 3 after immunisation, the mean temperatures of CpHV-1 inoculated goats were higher than the temperature of gE-negative BoHV-1 immunised goats (p < 0.001) (data not shown).

**Figure 1:**
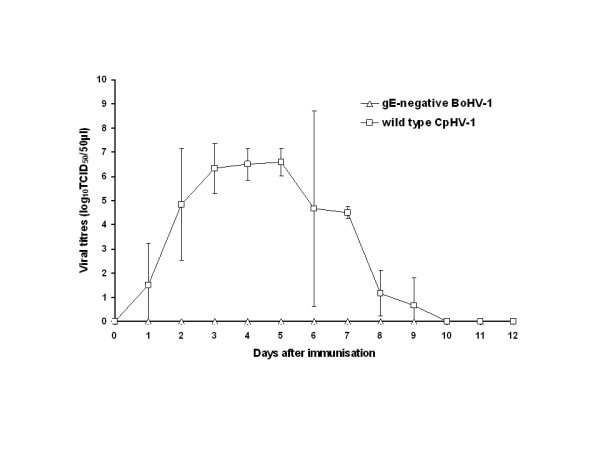
**Mean titres of BoHV-1 or CpHV-1 in nasal swabs recorded after intranasal immunisation of goats with either live attenuated gE-negative BoHV-1 vaccine or virulent CpHV-1 Ba-1**. Titres are expressed as log_10 _TCID_50 _per 50 μl of nasal secretions.

Following the first immunisation (day 0), only CpHV-1 was excreted by goats (Fig. 1). CpHV-1 was isolated in cell culture and was detected by PCR in nasal swabs but was not recovered from vaginal swabs and buffy coats. The peak viral titre was 10^6.6 ^TCID_50 _per 50 μl of nasal secretions. After the second immunisation (day 21), both samples from gE-negative BoHV-1 and from CpHV-1 infected goats were consistently negative by isolation in cell culture and detection by PCR.

### Viral excretion after CpHV-1 intravaginal challenge

**Figure 2:**
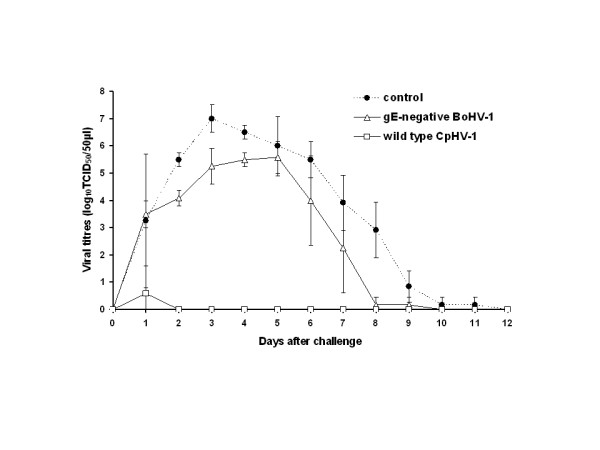
**Mean titres of CpHV-1 in vaginal swabs recorded after CpHV-1 Ba-1 intravaginal challenge of goats previously intranasally immunised with either live attenuated gE-negative BoHV-1 vaccine or virulent CpHV-1 Ba-1**. The control group was not inoculated with any preparation. Titres are expressed as log_10 _TCID_50 _per 50 μl of vaginal secretions.

Three weeks after the second immunisation, control and immunised goats were intravaginally challenged with the virulent CpHV-1 Ba-1 strain (Fig. 2). The mean CpHV-1 titres in vaginal swabs were significantly different (p < 0.0001) between groups immunised with either gE-negative BoHV-1 vaccine or virulent CpHV-1 and the non-immunised group. The lowest excretion titres were obtained in the CpHV-1 immunised group compared to gE-negative BoHV-1 immunised or non-immunised groups (p < 0.0001). The immunisation with gE-negative BoHV-1 vaccine decreased the mean challenge virus excretion titres: 1.42 log on day 2, 1.75 log on day 3, 1.5 log on day 6, 1.66 log on day 7, 2.75 on day 8, except on days 4, 5 and 9 after challenge where the mean excretion titres, although not significantly different, were lower than the mean excretion titres obtained on the same day in non-immunised goats. Furthermore, the gE-negative BoHV-1 vaccine shortened the challenge strain shedding. While no viral shedding was detected on day 12 in non-immunised group, goats from the gE-negative BoHV-1 immunised group excreted the challenge CpHV-1 during a significantly shorter time period. The shortest virus shedding, up to day 1 after challenge, was detected in the CpHV-1 immunised group (Fig. 2).

On days 1 and 4 after challenge, viruses from one goat in each group were further propagated individually and characterised using restriction enzyme analysis. The *Bst*EII profiles confirmed that viruses excreted after challenge by goats immunised with gE-negative BoHV-1 vaccine were CpHV-1 challenge Ba-1 strain (data not shown).

### Clinical protection against CpHV-1 intravaginal challenge

**Figure 3:**
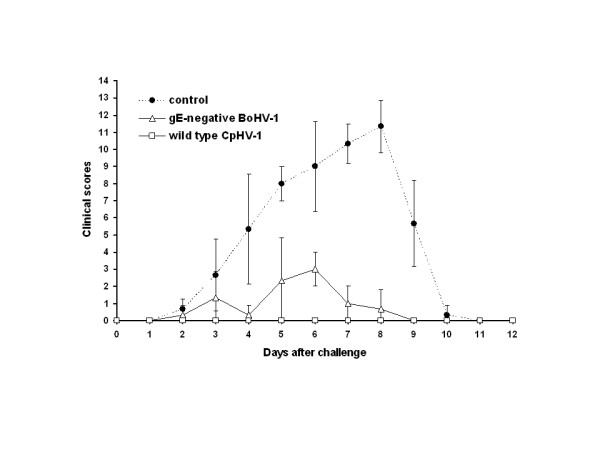
**Mean clinical scores recorded after CpHV-1 Ba-1 intravaginal challenge of goats previously intranasally immunised with either live attenuated gE-negative BoHV-1 vaccine or virulent CpHV-1 Ba-1**. The control group was not inoculated with any preparation.

Groups intranasally immunised with either virulent CpHV-1 or gE-negative BoHV-1 vaccines were protected against the clinical form of the genital CpHV-1 infection (Fig. 3). Consequently, the clinical score of each group was significantly lower (p < 0.0001) than in the non-immunised group. The statistical analysis revealed no significant difference between the gE-negative BoHV-1 immunised and the CpHV-1 immunised groups (p = 0.06) except at days 5 and 6 after challenge. At these days, goats immunised with gE-negative BoHV-1 vaccine showed mild oedema or vulva hyperemia.

The mean rectal temperature of immunised groups was significantly lower (p < 0.0001) than those of the non-immunised group except at the challenge peak (days 3 and 5 after challenge). Additionally, the group immunised by inoculation with virulent CpHV-1 exhibited a significant lower temperature (p < 0.0001) than the gE-negative BoHV-1 vaccine immunised group. Overall, goats did not show any sign of hyperthermia (data not shown).

### Immune responses after immunisation and challenge

**Figure 4:**
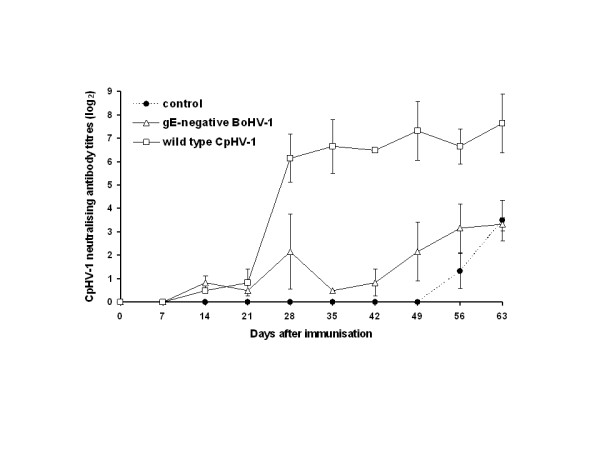
**Evolution of CpHV-1 neutralising antibody titres in goats intranasally immunised on day 0 with either live attenuated gE-negative BoHV-1 vaccine or virulent CpHV-1 Ba-1**. The control group was not inoculated with any preparation. Goats were intravaginally challenged with virulent CpHV-1 Ba-1 on day 42. Neutralising titres are expressed as the initial dilution of serum that neutralised 50% of wells, calculated using the Spearman-Kärber method.

In goats immunised with either virulent CpHV-1 or gE-negative BoHV-1 vaccine, CpHV-1 neutralising antibodies were observed from day 14 after the first immunisation (Fig. 4). No significant difference between groups was noticed. After the second immunisation, a sharp increase in CpHV-1 neutralising antibody titres was observed in the CpHV-1 immunised group. In contrast, goats immunised with gE-negative BoHV-1 did not show any boost of the primary immune response (Fig. 4). Following the CpHV-1 intravaginal challenge, the mean CpHV-1 neutralising antibody titres were significantly different (p < 0.0001) between groups immunised with gE-negative BoHV-1 or CpHV-1 and the non-immunised group. Interestingly, goats immunised with gE-negative BoHV-1 vaccine showed an increase in CpHV-1 neutralising antibodies, but their neutralising titres were much lower than that of CpHV-1 immunised goats (Fig. 4). Neutralising antibody titres were lower against BoHV-1 than CpHV-1 in all groups (data not shown). Moreover, all animals remained negative with the BoHV-1 gE blocking ELISA.

## Discussion

Taking advantage of the susceptibility of goats to BoHV-1 [32], the efficacy of a live attenuated gE-negative BoHV-1 vaccine was assessed in goats after two intranasal administrations followed by a subsequent CpHV-1 intravaginal challenge. The intranasal use of a gE-negative BoHV-1 vaccine enabled a cross-protection against CpHV-1 genital infection which is the natural route of infection in goats. As observed in human, nasal mucosa can serve as an efficient site for the induction of a specific protective response in the genital tract. It could be a consequence of the induction of specific IgA and IgG responses in vaginal secretions. The presence of specific antibody secreting cells (ASCs) in the genital tract have been demonstrated after nasal vaccination in mice [37,38]. In another study, it has been shown that T lymphocyte homing to the genital mucosa requires the interaction of integrins α_L_β_2 _and α_4_β_1 _with endothelial intercellular adhesion molecule-1 and vascular cell adhesion molecule-1 (VCAM-1), respectively [39,40]. Since both nasal and genital mucosa express VCAM-1, this adressin could be involved in the homing of specific ASCs to the genital tract. Moreover, chemokine like CC chemokine ligand 28, which is expressed in both tissues, could interact with the chemokine receptor 10 expressed on nasal ASCs and be involved too in the homing of specific ASCs to the genital area [41,42]. Although the underlying mechanism was not investigated in this study, it can be speculated that such pathways could be involved in the current protection.

Following the first immunisation, the safety of the gE-negative BoHV-1 vaccine in goats was evidenced by the absence of side effects and local or systemic reactions. Interestingly, goats did not excrete gE-negative BoHV-1 although a low level of BoHV-1 excretion was observed previously [32]. A weaker replication of gE-negative BoHV-1 could account for this result. Nevertheless, the presence of neutralising antibodies against BoHV-1 after the first and second immunisations suggests the replication of gE-negative BoHV-1 in goats. It can be hypothesised that the first immunisation induced a strong mucosal immunity leading to the neutralisation of newly replicated viruses after the second immunisation. The absence of detection of anti-gE antibodies in BoHV-1 gE blocking ELISA is consistent with the deletion of the gene encoding gE in the BoHV-1 vaccine. Moreover, such negative results in goats inoculated with CpHV-1 suggest a difference in the antigenicity of gE between BoHV-1 and CpHV-1.

Another issue to consider is the possible establishment of gE-negative BoHV-1 in a latent state in vaccinated goats. Indeed, BoHV-1 is able to establish latency in goats but with a poor reactivation success rate [30,31]. However, gE-negative BoHV-1 is less effective in reactivation and reexcretion than wild type viruses in calves [4,43]. Therefore, the risk of reactivation and reexcretion of a gE-negative BoHV-1 in goats is low. On the other hand, the vaccination could also lead to the emergence of new recombinant viruses. Indeed, despite the fact that in the subfamily *Alphaherpesvirinae*, viruses of different species show very few sequence similarities to allow homologous recombination, several interspecific recombinants were isolated *in vitro *[44]. Natural recombinants between equid herpesviruses 1 and 4 were, for example, recently identified [45]. Therefore, the question of recombinants rising from cross-infection of CpHV-1 infected goats with BoHV-1 needs to be considered. Among the cluster of ruminant alphaherpesviruses related to BoHV-1, only two recombinant viruses between BoHV-1 and BoHV-5 were isolated, and no recombinant between BoHV-1 and less closely related CpHV-1 and CvHV-2 was detected *in vitro *[46]. Consequently, in regards of these data and especially the low level of excretion, the vaccination described here is likely to be completely safe.

The reduction of the clinical score was considered as the most relevant parameter showing the efficacy of nasal immunisation against CpHV-1. Goats immunised intranasally with gE-negative BoHV-1 vaccine were clinically protected. Moreover, the difference between goats immunised with either gE-negative BoHV-1 or CpHV-1 was not statistically significant. The gE-negative BoHV-1 vaccine was not only effective in preventing development of genital disease upon challenge, but also in significantly reducing the magnitude and the duration of challenge CpHV-1 excretion. A high protection against clinical signs was also observed after immunisation by intranasal infection with CpHV-1. However, in natural conditions, the same kind of protection is not likely to be reached because the main route of transmission is venereal instead of respiratory [11,12]. The current CpHV-1 vaginal challenge used in this assay was even more severe than in previous experiments [47,48] and this result brings a reliable validation of the current study. The significant differences observed between immunised and non-immunised groups have been obtained with a relatively low number of animals, therefore despite a lower power of the statistical test. These data allowed the identification of a significant effect of vaccination with the live attenuated gE-negative BoHV-1 vaccine [49]. Moreover, nasal vaccination is an interesting alternative for inducing specific antibody responses in female genital tract, both for convenience and because the outcome of vaginal vaccination might be dependent on the time point in the oestral cycle for vaccine administration [35].

Concerning infection control, such vaccination could bring several advantages. Indeed, the existence of antigenic cross-reactions between ruminant alphaherpesviruses related to BoHV-1 and their ability to cross the species barrier raise theoretical problems for the differential diagnosis and the detection of any other virus reservoir, both in regions and countries where BoHV-1 infection has been eradicated and in those where the control of IBR is currently or will be undertaken [2]. The use of such vaccination could reduce the circulation of CpHV-1 in goats which would be therefore less involved in BoHV-1 misdiagnosis due to infection with a closely related alphaherpesvirus. Moreover, the development of new vaccines in order to protect minor species against infection causing economical and management problems meets a poor interest from the pharmaceutical industry. In this context, a classical inactivated vaccine inducing a good protection against CpHV-1 infection in goats was developed but was not licensed [21,22]. Consequently, it was required to investigate the capacity of an already licensed bovine vaccine to induce a cross-protection against a related virus infection in goats according to the principle of the cascade. The European Union has recently pointed out the requirements of medicinal veterinary products for minor uses and minor species, as goats for example [50]. The results obtained in this study clearly show that a bovine vaccine can be safely and efficiently used in goats.

## Conclusion

Regarding the issue of ruminant alphaherpesvirus diagnosis, the economical constraints of the veterinary pharmaceutical industry and the well-being of animals, this study brings an expected tool for the CpHV-1 induced disease prevention. Indeed, the intranasal administration of a live attenuated gE-negative BoHV-1 vaccine protects goats clinically and virologically against CpHV-1 genital infection which is the natural route of infection in goats. In addition, the current study emphasises the interest of studying intranasal vaccination approaches against genitally transmitted infections through the mucosal immune system.

## Methods

### Cells and viruses

The Madin-Darby bovine kidney (MDBK) cell line (ATCC CCL22) was maintained in Dulbecco-Minimal Essential Medium (D-MEM) supplemented with 10% of foetal bovine serum (FBS). The challenge CpHV-1 Ba-1 strain [51] was produced by infection of MDBK cells in D-MEM supplemented with 10% of FBS. At 72 h after infection, culture medium was removed and clarified by centrifugation at 1,500 × g for 20 min. Supernatants were divided into aliquots, frozen at -80°C and titrated by tissue culture infectious dose 50 (TCID_50_) method on MDBK cells. The gE-negative BoHV-1 vaccine virus strain used for immunisation is the commercial vaccine Rispoval^® ^IBR-marker vivum (Pfizer Animal Health). The BoHV-1 Iowa [52] and the CpHV-1 Ba-1 strains were used for serum neutralisation assays.

### Experimental design

Nine dairy Alpine, Ionica, Maltese and Saanen crossbred goats, approximately 4–5 years of age, were used. All goats were originated from a CpHV-1 seronegative flock in Italy. Prior to inoculation, absence of antibodies against BoHV-1 and CpHV-1 was confirmed by serum neutralisation assay. The goats were randomly divided in three groups of three goats. Each group was separated in different airspaces. Two groups were immunised intranasally by aerosolization twice three weeks apart as follows: group 1 received 2 ml per nostril of virulent CpHV-1 Ba-1 at a dose of 10^5.25 ^TCID_50_/50 μl, and group 2 received 2 ml per nostril of gE-negative BoHV-1 vaccine at a dose of 10^4.25 ^TCID_50_/50 μl. Group 3 served as negative control and was kept uninoculated before challenge. On day 42, all goats were challenged by the intravaginal route with 4 ml of virulent CpHV-1 Ba-1 (10^6.25 ^TCID_50_/50 μl). All precautions were taken to avoid viral spread. Clothes and boots were changed before entering any stable. For handling, new gloves were used between groups. Clinical monitoring and all sampling procedures were carried out in a blind manner. Goats were clinically examined daily from day -1 (before infection) up to day 21 following the challenge and rectal temperatures were also measured up to day 16 post-challenge. Clinical observations were carried out at approximately the same time everyday and by the same scientist throughout the study. Clinical monitoring included the following clinical signs: depression, anorexia, vaginal haemorrhage, vaginal discharge, pain, hyperemia of vulva and vagina, oedema of vulva and vagina, number of lesions in vulva and vagina. A clinical score from 0 to 2 was given for each clinical parameter except temperature. Scores quantifying the oedema of vulva and vagina were multiplied by 2. Scores quantifying the number of lesions in vulva and vagina were multiplied by 3. Blood samples for serology were collected from the jugular vein of animals weekly during the whole experiment at days 0, 7, 14, 21, 28, 35, 42, 49, 56 and 63 after the primary immunisation. Serums obtained after centrifugation were stored at -20°C until analysis. Heparinised blood samples for buffy coat extraction, nasal and vaginal swabs were collected daily up to day 14 after first immunisation, up to day 14 after second immunisation, and during 14 days post-challenge, using one swab per animal, swabbing deeply into each nostril or vagina. The experiment was carried out following national and international guide for the care and use of experimental animals.

### Viral characterisation

Samples were immersed in 1 ml of D-MEM and centrifuged at 5,000 × g for 5 min. The supernatant was then treated with a 10% antibiotics mixture (5,000 UI/ml penicillin, 2,500 μg/ml streptomycin, 10 μg/ml amphotericin) for 30 min at room temperature and titrated by the TCID_50 _method on MDBK cells cultured in 96-well microtitre plates. The excess of samples was stored frozen at -80°C. Cells were examined daily for cytopathic effect (CPE). The virus titre was expressed as TCID_50 _per 50 μl of secretion. Buffy coats were separated from blood by centrifugation in presence of lympholyte (Cedarlane, Canada). Sample preparation and polymerase chain reaction (PCR) were performed as previously described with minor modifications [13,53]. Viral DNA were prepared from supernatants of MDBK cell cultures infected with viruses isolated on days 1 and 4 after challenge [16]. Two μg of DNA were submitted to *Bst*EII restriction analysis (New England Biolabs) and DNA fragments were separated in a 0.7% Tris Acetate EDTA gel for 22 h at 30 V/cm and 500 mA.

### Serological analysis

Serial twofold dilutions of each serum were mixed with either 100 TICD_50 _of BoHV-1 Iowa strain or 100 TICD_50 _of CpHV-1 Ba-1 strain in 96-well microtitre plates. The plates were held for 90 min at room temperature and 20,000 MDBK cells were then added to each well. Analysis was done after three days of incubation at 37°C in presence of 5% CO_2_. The titre of each serum was expressed as the highest serum dilution which neutralised the virus in 50% of the wells [43]. The BoHV-1 gE blocking ELISA (Herdchek Anti-IBR gE, Idexx, Germany) was used following the manufacturer instructions. Serums were analysed in duplicate.

### Statistical analysis

Statistical comparisons in the clinical, virological and serological data were performed in the form of mixed models for repeated measurements by SAS procedure (procedure MIXED) [54].

## Authors' contributions

JT, MT, ETh and CB designed the experiments and analysed the data. JT, MT, MC and ETa performed the experiments. JT, MT, ETh and CB drafted the manuscript together. BM and FM helped to draft the manuscript. All authors read and approved the final manuscript.
